# Supplementary material for: Computer simulations of food oral processing to engineer teeth cleaning
Source: Nat Commun. 2019 Aug 8;10:3571. doi: 10.1038/s41467-019-11288-5 (PMC6687884; doi:10.1038/s41467-019-11288-5)
Supplement: Supplementary file 1 — Supplementary information [file 41467_2019_11288_MOESM1_ESM.pdf]

Computer simulations of food oral processing to engineer teeth cleaning

Skamniotis et al.

SUPPLEMENTARY INFORMATION

Supplementary discussion 1

This discussion concerns the effect of bite speed, $\dot{\delta}$, on the mandible force-displacement, $F - \delta$, numerical predictions for $\dot{\delta} = 1.66$ mm s^{-1}, $\dot{\delta} = 16.6$ mm s^{-1} and $\dot{\delta} = 166.6$ mm s^{-1} (for fixed $\mu = 0.3$). The results are depicted in supplementary Figure 1 (the available experimental data for $\dot{\delta} = 1.66$ mm s^{-1} and $\dot{\delta} = 16.6$ mm s^{-1} are also plotted). Performing simulations at lower rates i.e. $\dot{\delta} = 0.16$ mm s^{-1}, is impractical because the associated bite durations become exceedingly long for an Explicit FE integration, which may also give rise to numerical errors [1]; moreover, values of $\dot{\delta} \leq 0.16$ mm s^{-1} are unrealistic to chewing [3]. On the other hand, simulations for $\dot{\delta} > 166.6$ mm s^{-1} were not performed, since the associated applied strain rates would exceed considerably the range $0.0001 - 5$ s^{-1}, based on which the constitutive law was calibrated. The good experimental-FE model agreement for $\dot{\delta} = 1.66$ mm s^{-1} and $\dot{\delta} = 16.6$ mm s^{-1} supports that the FE model is capable of predicting the chewing response accurately at different speeds. The experimental-FE model discrepancy seen for $\dot{\delta} = 1.66$ mm s^{-1} beyond the onset of breakdown ($\delta > \delta_o$), is due to the fact that the experimental $G_c = 0.93$ kJ m^{-2} value was obtained for crack speeds (average 5 mm s^{-1}) significantly higher than the ones applied in the model (estimated to be within $0.5 - 1$ mm s^{-1} for $\dot{\delta} = 1.66$ mm s^{-1}). As a result, the breakdown resistance of the food is over-predicted. This indicates that modelling rate dependency in G_c may be necessary for predicting the chewing response for bite speeds of different orders of magnitude.

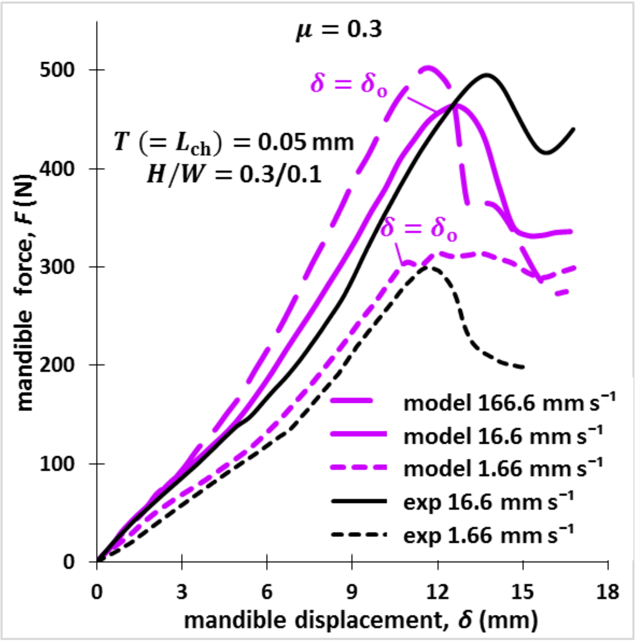

Supplementary Figure 1. Effect of bite speed on the mandible force-displacement predictions. Speeds of 1.66 mm s^{-1}, 16.6 mm s^{-1} and $\dot{\delta} = 166.6$ mm s^{-1} are compared for a fixed friction coefficient of 0.3; the experimental data for the speeds 1.66 mm s^{-1} and 16.6 mm s^{-1} are also plotted.

Supplementary discussion 2

This discussion concerns the sensitivity of the numerical $F - \delta$ results for $\dot{\delta} = 16.6$ mm s^{-1} and $\mu = 0.3$, upon the characteristics of the FE mesh. Mesh dependency in the current mastication model can be induced by three main mechanisms. One is through variation in the characteristic length of each element, $L_{\mathrm{ch}}(\approx T)$, based on which the damage computations are mainly performed; the latter principally influences the crack propagation results, such that these may not converge into a true response with mesh refinement [2]. The second mechanism of mesh dependency relates to insufficient discretisation level i.e. when the elements are too large in size (coarse mesh). The third concerns the element type-shape and specifically for the hexahedral elements used here, the element aspect ratio, $\mathcal{A}$, with respect to the direction of maximum absolute strain (here it is the direction of teeth indentation).

Regarding element type-shape, the superiority of hexahedral elements versus tetrahedral elements for this application, is already established [1]; also note that multiple integration points within elements were not employed as this would have led to a prohibitive computational cost. Furthermore, in order to reduce element distortion at large indentations, an optimised element aspect ratio value of three has also been previously proposed [4]; a fixed $\mathcal{A} = H/W \approx 3$ is therefore used throughout the following mesh sensitivity analysis.

The effect of L_{ch} and element size, is investigated (for $\mu = 0.3$) in two steps. Firstly, a fixed element thickness, $T = 0.05$ mm, is uniformly used along the length of fine mesh region in the food item, while the average element size is scaled by varying the element dimensions H, W to satisfy the following H/W ratios: 0.15/0.05, 0.3/0.1, 0.45/0.15, 0.6/0.2. Thus, in this step, the effect of element size i.e. discretisation level is isolated, since food separation is not affected significantly due to a constant $L_{\mathrm{ch}} \approx T = 0.05$ mm. The corresponding results are illustrated in supplementary Figure 2(a), which shows that within the studied H/W range, the $F - \delta$ response is insensitive to mesh density up to the onset of breakdown (at $\delta = \delta_{\mathrm{o}}$). For $\delta > \delta_{\mathrm{o}}$, however, a $H/W = 0.6/0.2$ leads to an under-predicted breakdown resistance i.e. force drops steeply, while the other three H/W ratios continue to give similar predictions, adding credibility to the mesh design employed in the chewing model (corresponding to H/W= 0.3/0.1). The fact that a H/W= 0.3/0.1 reduced the analysis time by approximately 50% compared to H/W= 0.15/0.05, explains the reason for using H/W = 0.3/0.1 throughout the study.

Now for fixed $H/W = 0.3/0.1$ (and $\mu = 0.3$), the individual effect of L_{ch} on the results is investigated by varying T between: 0.02 mm, 0.05 mm, 0.1 mm, 0.2 mm. In the regime $\delta > \delta_{\mathrm{o}}$ supplementary Figure 2(b) shows that the food breakdown resistance increases (smoother drop in force) with decreasing L_{ch}. Similar phenomena have been reported in a recent study [2], and have been attributed to the fact that very small elements must deform exceedingly in order to reach the required displacement, u_{f}, postulated by the G_{f} criterion (see Equation (7)); this affects the response of elements adjacent to the ones undergoing damage, leading to crack tip mesh distortion effects of which the severity varies depending on the mesh density. These effects however can be reduced by regulating the crack tip deformation field between different mesh densities, through the numerical correction strategy described extensively in [2]. Therefore, in order to reduce the mesh dependency shown here in supplementary Figure 2(b), the same strategy is applied. It involves using a strain energy density dissipation parameter, U_{c} (kJ m^{-3}), in Equation (11), such that G_{f} is now defined by:

$$G_{\mathrm{f}} = R_{\sigma} R_{\varepsilon} \frac{U_{\mathrm{c}}}{2} L_{\mathrm{ch}} \qquad (S1)$$

where U_{c} is defined as [2]:

$$U_{\mathrm{c}} = \frac{G_{\mathrm{c}}}{L_{\mathrm{ch}_*}} \qquad (S2)$$

where L_{ch_*} is the specific characteristic element length which leads to the best agreement with experimental crack propagation data when G_{f} is defined by Equation (11), i.e. when the above strategy is not used. The crack propagation data are obtained from an independent tensile fracture experiment; for this material, this was performed in [2] and gave $L_{\mathrm{ch}_*} = 0.05$ mm (and thus $U_{\mathrm{c}} = 9.3$ kJ m^{-3} since $G_c = 0.93$ kJ m^{-2}). Therefore, note that the FE results presented in Figures 4−6 remain practically the same when the U_{c} parameter is used, since these correspond to $L_{\mathrm{ch}} = L_{\mathrm{ch}_*} = 0.05$ mm; for this reason the above strategy was not necessary for studying food breakdown and cleaning efficacy. Nevertheless, when $L_{\mathrm{ch}} \neq 0.05$ mm, supplementary Figure 2(c) shows that using the U_{c} parameter has merits in providing more accurate results compared to supplementary Figure 2(b), such that mesh dependency is significantly reduced. Specifically, supplementary Figure 2(c) demonstrates that reasonable breakdown predictions are obtained for any $T(=L_{\mathrm{ch}})$ in the range 0.02−0.1 mm. Furthermore, note that the two extremes, $T = 0.02$ mm and $T = 0.2$ mm, are not in fact reasonable values to be used in this model but instead are used for the purpose of this mesh sensitivity study. $T = 0.02$ mm leads to an extremely large mesh size of 1864660 elements, compromising computational efficiency, while $L_{\mathrm{ch}} = 0.2$ mm influences the element aspect ratio, H/T, degrading the performance of the mesh at large teeth indentations. Instead, reasonable values lie in the L_{ch} range 0.05−0.1 mm, for which the mesh dependent effects are very small (see supplementary Figure 2(c)), thus adding credibility to the model. The same L_{ch} range of 0.05−0.1 mm was also found to give accurate fracture predictions for this material both in the single-edge-notched-tensile geometry studied in [2], as well as in the cylindrical indentation geometry reported in [4]. This agreement between different fracture geometries adds further credibility to our numerical modelling methodology.

Regarding potential mesh orientation effects on the results, these are assumed here to be negligible. Specifically, although in Figures 4(c)&(e) the major crack faces in the food item have been shown to approximately coincide with the original plane of element facets, this is triggered by the fact that the original plane of element facets is normal to the direction of the applied maximum tensile strain, ε_1 (which causes separation into two pieces). It has been generally observed that the morphology of the main crack faces may be influenced by several factors, one of these being the ratio between G_{c} and the overall stress levels applied in the food (which depends on $\dot{\delta}$). As a result, although the major crack faces aligned significantly with the original plane of element facets for the conditions $L_{\mathrm{ch}} = 0.05$ mm, $H/W = 0.3/0.1$, $\dot{\delta} = 16.6$ mm/s, this is not true when other conditions are simulated e.g. when $L_{\mathrm{ch}} = 0.05$ mm, $H/W = 0.3/0.1$, $\dot{\delta} = 166.6$ mm/s. Stronger evidence of arbitrary crack predictions by using the current damage scheme can be found in [4]; the former study demonstrated cracking inclined at 45° with respect to the original plane of the element facets. The above lead to the conclusion that the orientation of the mesh does not necessarily predefine the crack path, indicating that our model is a powerful tool for predicting complex fracture patterns during food oral processing.

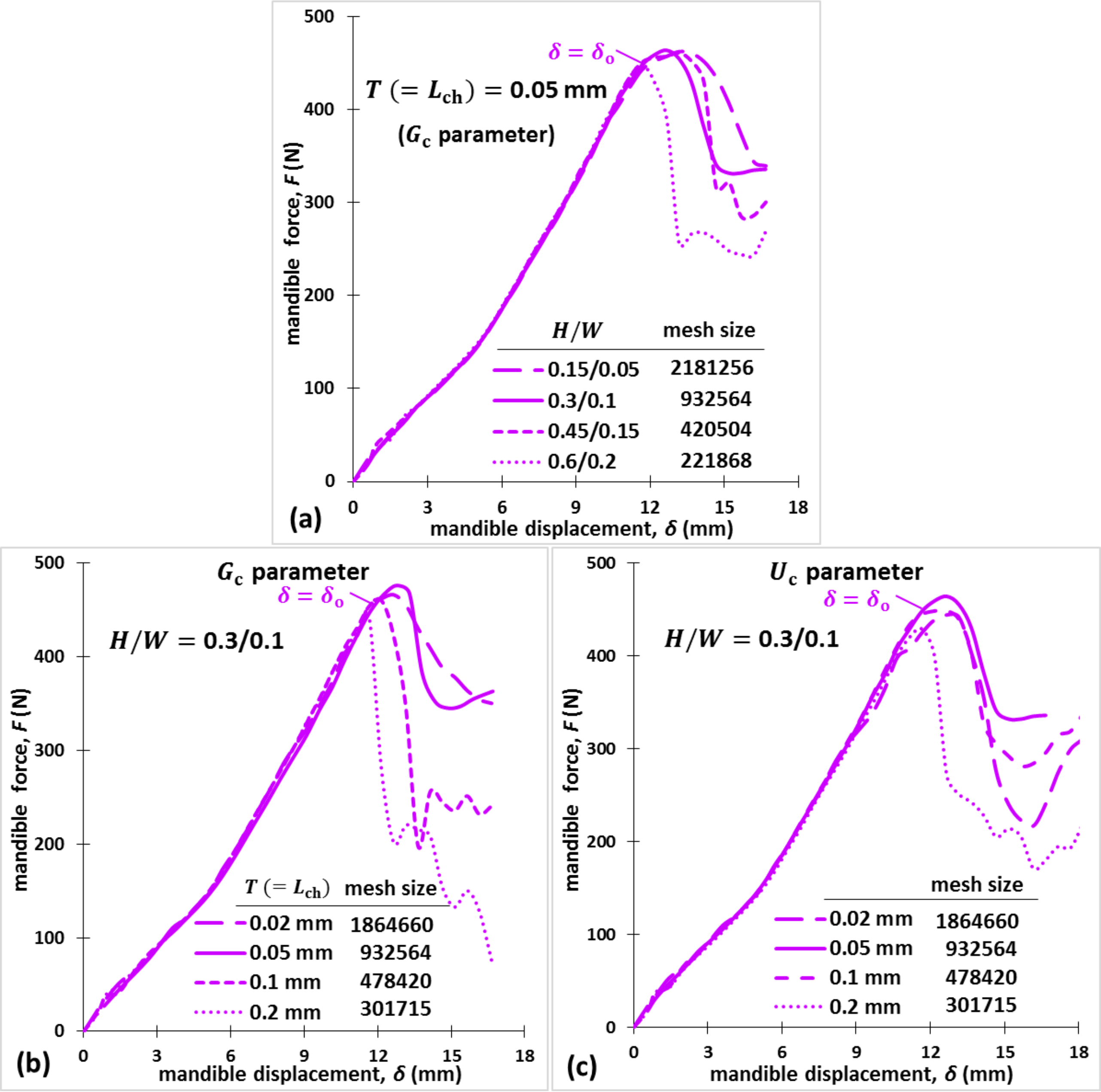

Supplementary Figure 2. Summary of mesh sensitivity analysis results. (a) shows mandible force-displacement predictions between using four different element average dimensions, H/W = 0.15/0.05, 0.3/0.1, 0.45/0.15, 0.6/0.2, in the food item, while keeping the thickness T (corresponding to the direction of the food specimen length) of all the elements in the fine mesh region fixed at 0.05 mm (for the given direction of cracking here the thickness is practically equal to L_{ch}); the G_c parameter was used in Equation (11). (b) shows corresponding results for consistent average element dimensions, $H/W = 3$, while varying the thickness of the all the elements in the fine mesh region between: 0.02 mm, 0.05 mm, 0.1 mm, 0.2 mm, and using G_c in Equation (11). (c) shows the same results as in (b) when the numerical strategy against mesh dependency described in [2] is applied i.e. when a term $U_c L_{ch}$ is used in Equation (11) instead of the parameter G_c. For each condition the corresponding mesh size (number of elements) is indicated, while a friction coefficient of 0.3 is used throughout.

Supplementary references

1. Simulia, A.V., *6.13 Documentation.* Dassault systemes, 2013.
2. Skamniotis, C., M. Elliott, and M. Charalambides, *On modelling the constitutive and damage behaviour of highly non-linear bio-composites–mesh sensitivity of the viscoplastic-damage law computations.* International Journal of Plasticity, 2018.
3. Swackhamer, C. and G.M. Bornhorst, *Fracture properties of foods: Experimental considerations and applications to mastication.* Journal of Food Engineering, 2019.
4. Skamniotis, C., M. Elliott, and M. Charalambides, *On modeling the large strain fracture behaviour of soft viscous foods.* Physics of Fluids, 2017. **29**(12): p. 121610.
